# Validation of the Developed Zero-Order Infrared Spectrophotometry Method for Qualitative and Quantitative Analyses of Tranexamic Acid in Marketed Tablets

**DOI:** 10.3390/molecules26226985

**Published:** 2021-11-19

**Authors:** Nerdy Nerdy, Linda Margata, Bunga Mari Sembiring, Selamat Ginting, Effendy De Lux Putra, Tedy Kurniawan Bakri

**Affiliations:** 1 Department of Pharmacy, Faculty of Pharmacy, Institut Kesehatan Deli Husada Deli Tua, Deli Tua Timur, Deli Tua, Deli Serdang 20355, Indonesia; lindamargata@hotmail.com; 2 Department of Public Health, Faculty of Public Health, Institut Kesehatan Deli Husada Deli Tua, Deli Tua Timur, Deli Tua, Deli Serdang 20355, Indonesia; bungamerisembiring@gmail.com (B.M.S.); selamatginting18@gmail.com (S.G.); 3 Department of Pharmacy, Faculty of Pharmacy, Universitas Sumatera Utara, Padang Bulan, Medan Baru, Medan 20155, Indonesia; effendy@usu.ac.id; 4 Department of Pharmacy, Faculty of Mathematics and Natural Sciences, Universitas Syiah Kuala, Kopelma Darussalam, Syiah Kuala, Banda Aceh 23111, Indonesia; tedykbakri@unsyiah.ac.id

**Keywords:** validation, infrared spectrophotometry, qualitative, quantitative, tranexamic acid

## Abstract

(1) Background: The functional groups present in tranexamic acid allow direct infrared detection analysis. This study aimed to develop, apply, and validate an infrared spectrophotometry method used for qualitative and quantitative analyses of tranexamic acid in marketed tablets. (2) Methods: This was a descriptive observational study that consisted of several stages: determining the specific wavenumber for analysis, obtaining a simple linear regression equation, analyzing tranexamic acid both qualitatively and quantitatively, and validating the developed method for routine analysis. (3) Results: The peak analysis obtained a range of baseline wavenumbers from 1679.17 to 1295.25 cm^−1^. The regression equation obtained was Y = 310.8527 × X + 0.9718, and the coefficient of determination (R^2^) obtained was 0.9994. The tranexamic acids in marketed tablets overall have a similarity index value of more than 0.90 and overall have levels ranging between 97.0% and 103.0%. The infrared spectrophotometry method that was successfully developed, applied, and validated for qualitative and quantitative analyses of tranexamic acid in marketed tablets meets the requirements both qualitatively and quantitatively of the tablet monograph. (4) Conclusions: The infrared spectrophotometry method has been validated and meets the requirements for accuracy, precision, detection limit, quantitation limit, linearity, range, and specificity.

## 1. Introduction

Hemostasis is the body’s natural process of stopping bleeding either spontaneously or not spontaneously [1]. Bleeding in the body stimulates the body’s natural mechanisms to clot the blood to stop bleeding [2]. This condition is the cause of pathological conditions and requires therapy, which is tranexamic acid [3]. Tranexamic acid is a lysine-derived synthetic antifibrinolytic that acts by reversibly blocking the lysine-binding region on the plasminogen molecule [4]. This medicine should be used according to a doctor’s prescription and in the short term. It prevents side effects and organ damage, such as kidney and bone marrow damage [5,6]. Tranexamic acid is marketed in a tablet dosage form available in strengths of 500 mg per tablet [7]. Drugs marketed must meet aspects of efficacy, quality, and safety [8]. The finished medicinal product must meet the quality requirements stated in the monograph before being marketed [9].

Several previous studies for qualitative and quantitative analyses of tranexamic acid have been conducted. An analysis using ultraviolet spectrophotometry was carried out with various specific reaction treatments [10], and an analysis using infrared spectrophotometry was carried out using first order derivatization treatment [11]. Meanwhile, high-performance liquid chromatography analysis with various detectors can be applied to analyze tranexamic acid with or without treatment [12,13,14,15]. Several previous reports about tranexamic acid analyses are shown in Table 1.

**Figure 1:**
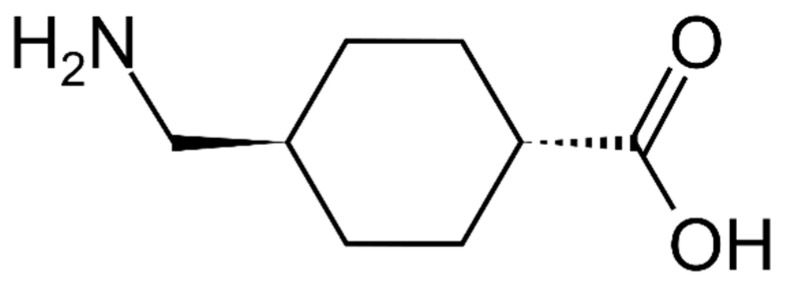
Molecular structure of tranexamic acid.

The analytical research on tranexamic acid is limited because it lacks adequate chromophore and auxochrome groups. It is not easy to analyze using ultraviolet–visible spectrophotometry [16]. High-performance liquid chromatography is a complex method, requires a high cost, and cannot be used to analyze all samples [17]. To overcome the drawbacks of high-performance liquid chromatography analysis, an alternate technique for qualitative and quantitative tranexamic acid analysis must be developed. Figure 1 presents the molecular structure of tranexamic acid. Given that tranexamic acid is the medicine of choice for bleeding, it necessitates a quick and valid examination to guarantee that it fits qualitative and quantitative preparation standards [18,19].

Infrared spectrophotometry measurements have the advantages of environment-friendliness, simplicity of sample preparation, ease of operation, and short analysis time. Infrared spectrophotometry is widely used to analyze pharmaceutical products, environmental substances, and biological fluids [20]. Organic analysis can be performed, or potassium bromide pellets can be used. The researchers chose the potassium bromide pellet approach because it is less harmful to the environment than organic solvents. Previous research identified other active compounds using the potassium bromide pellet approach and the zero-order method. However, the use of tranexamic acid in tablet forms has never been investigated or verified [21].

Functional groups present in tranexamic acid are used for analysis by the infrared spectrophotometry method. Infrared spectrophotometry application in the pharmaceutical industry is very popular as a tool for qualitative analysis (identification) and quantitative analysis (determination) because of its good sensitivity [22]. Researchers are therefore encouraged to use the potassium bromide pellet and zero-order procedures to quantify tranexamic acid levels and confirm the analytical method considering this background.

The laboratory requires method validation because it can determine the level of confidence resulting from an analytical method. The validation findings of the analytical technique can be used as a reference to establish the method’s validity so that it can be utilized in the routine analysis [23]. Revalidation should be done, even though the previous validation produced data following the acceptance criteria. The differences of differences in the equipment, reagents, analysts, or time will affect the sensitivity [24].

Based on the information presented above, the researchers were interested in establishing a direct analysis of the infrared spectrophotometry technique (zero-order without derivatives) as an alternative approach for evaluating tranexamic acid levels that was fast, straightforward, simple, and green. The method developed was then applied to determine the levels of tranexamic acid in marketed tablets. Validation of the analytical methods was also carried out to prove the validity of the analytical method before it is recommended for application in the pharmaceutical industry or government institutions as a quality-control method.

## 2. Results

### 2.1. Preliminary Study of Infrared Spectrophotometry

**Figure 2:**
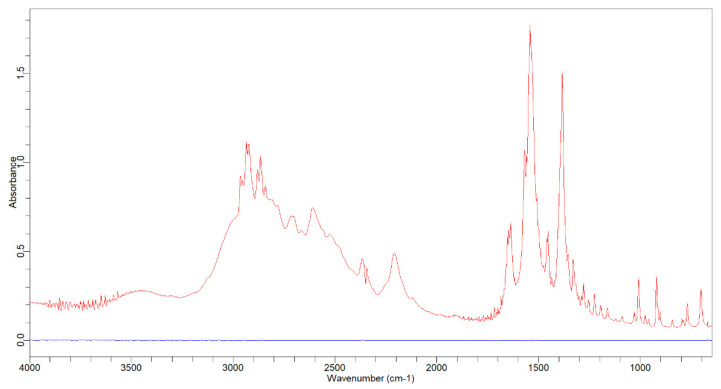
Overlay spectrum of potassium bromide (blue line) and tranexamic acid (red line).

**Figure 3:**
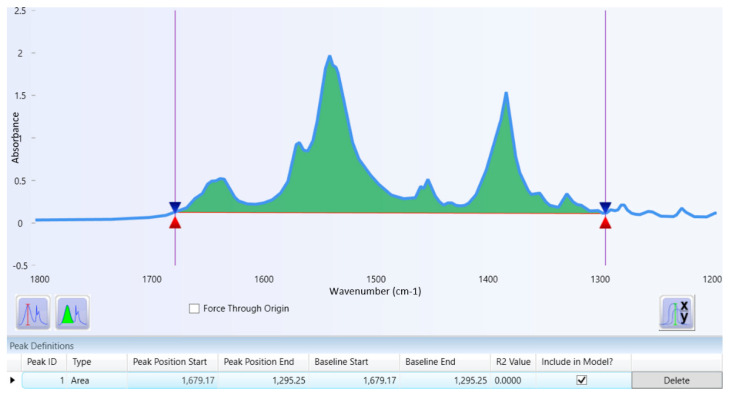
Baseline and area for quantitative analysis of tranexamic acid.

Figure 2 illustrates the overlay spectrum of potassium bromide (blue line) and tranexamic acid with a potassium bromide mixture (red line). The tranexamic acid spectrum was analyzed to obtain the specific wavenumbers and functional groups of tranexamic acid. Table 2 presents the specific wavenumbers and functional groups of tranexamic acid. Figure 3 illustrates the baseline and area for quantitative analysis of tranexamic acid.

The infrared spectrum, as shown in Figure 2, from potassium bromide (blue line) does not provide any absorption in the measurements (after background measurements with potassium bromide)—the specific wavenumbers are in Table 2.

### 2.2. Regression of Tranexamic Acid

**Figure 4:**
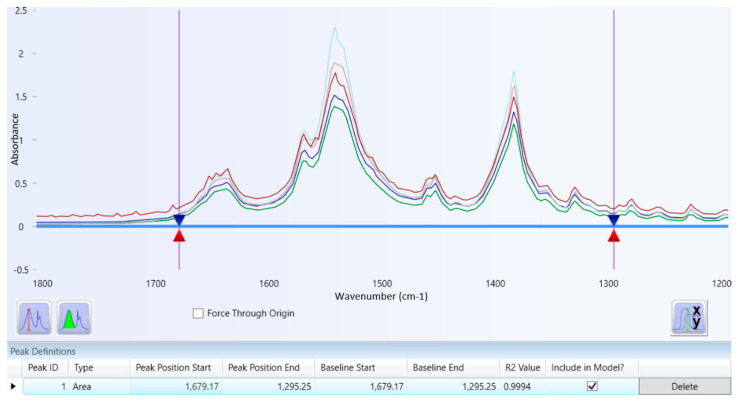
Overlay spectrum of regression data set series of tranexamic acid.

**Figure 5:**
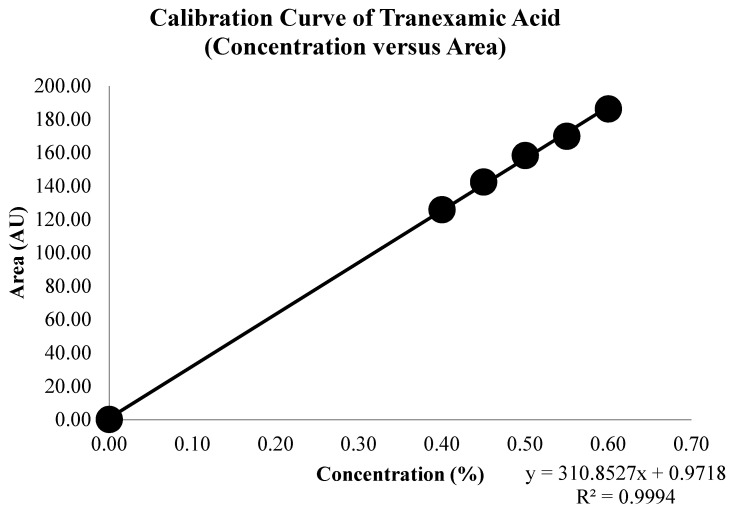
Calibration curve of tranexamic acid.

A set of tranexamic acid concentrations was measured and analyzed to obtain a series of tranexamic acid areas. Figure 4 illustrates the overlay spectrum of tranexamic acid regression data set series. The concentration and area series from the tranexamic acid regression data set and the tranexamic acid calibration curve are shown in Table 3 and Figure 5, respectively. The regression equation and coefficient of determination were constructed using the series concentration and area from the tranexamic acid regression data set acquired from the analysis findings in Table 3. The regression equation obtained was y = 310.8527x + 0.9718, and the coefficient of determination (R^2^) obtained was 0.9994, as illustrated in Figure 5.

### 2.3. Analysis of Tranexamic Acid in Marketed Tablets

Table 4 presents the results for qualitative and quantitative analyses of tranexamic acid in marketed tablets.

## 3. Discussion

### 3.1. Preliminary Study of Infrared Spectrophotometry

This investigation started with background measurements using potassium bromide pellets and then the combination of potassium bromide pellets and tranexamic acid in a pellet form was observed to see how potassium bromide affected tranexamic acid analysis. These results prove that potassium bromide does not interfere with the tranexamic acid analysis. The infrared spectrum of tranexamic acid (red line) in Figure 2 provides an absorption response similar to that in the literature, both in terms of spectrum and specific wave numbers [25]. Tranexamic acid was quantified using three primary peaks in the fingerprint area with wavenumbers ranging from 1800 to 650 cm^−1^, notably wave numbers 1638.2, 1541.3, and 1384.7 cm^−1^ [26,27,28].

The addition of tranexamic acid standard spectrum performed a qualitative analysis of tranexamic acid into the system library. Infrared spectrophotometry provides a computerized facility for qualitative analysis by matching unknown sample spectrums against spectrum data contained in the system library. This action resulted in the similarity between the sample spectrum that is qualitatively unknown and the standard spectrum in the system library [29]. The particular wavenumbers in Table 2 obtained from the tranexamic acid analysis illustrate the proximity to the wavenumbers in the literature and the related functional groups present in the molecular structure of tranexamic acid. [30].

The fingerprint capability of infrared spectrophotometry offers the convenience of analyzing the unique chemical characteristics of each compound in the sample. The procedures used in this research have been able to ensure the homogeneity and consistency of the results. Before pressing, the tranexamic acid and potassium bromide combination was weighed in equal parts (40 mg). Furthermore, pressing was done with the same tonnage (2.0 tons) and at the same time (2 min). It is feasible to achieve a homogenous film thickness with homogeneity in the weight of the tested mixture, pressing tonnage, and pressing duration [31].

A quantitative analysis was carried out on peak area analysis by calculating the sum area of the three main peaks. The technique for obtaining the peak area is typical under the band area, and it begins with identifying the baseline and concludes with determining the area. The peak baseline analysis obtained a range of baseline wavenumbers from 1679.17 to 1295.25 cm^−1^. In that wavenumber, the peak area of the three main peaks will be completely covered, as shown in Figure 3. The peak baseline is the lowest point on both sides of the peak spectrum to be analyzed. The peak area is the area covered by the peak and baseline spectrum. Quantitative analysis using the peak area provides better accuracy and precision and avoids an analysis error using peak absorbance or peak height [32].

### 3.2. Regression of Tranexamic Acid

Quantitative analysis using infrared spectrophotometry utilizes regression data sets that will produce a regression equation. A greater tranexamic acid content in the pellets resulted in a larger tranexamic acid area, as shown in Figure 4. In accordance with the Lambert-Beer law, which follows in the quantitative analysis by infrared spectrophotometry, it can be assumed that there is a linear relationship between the concentration of the compound and the response (absorbance or height or area) produced by the compound [33].

The regression equation obtained can analyze the concentration of tranexamic acid in a tablet with an unknown concentration. Good regression data sets have a coefficient of determination that is more than 0.99, indicating that the concentration can be determined from the area using the regression equation [34].

### 3.3. Analysis of Tranexamic Acid in Marketed Tablets

Analysis of tranexamic acid in marketed tablets was performed using the developed infrared spectrophotometric method. Measurements were made of the area with a predetermined baseline. Qualitative analysis with the tranexamic acid similarity index parameter and quantitative analysis with the tranexamic acid level parameter were carried out.

The findings of the qualitative analysis of tranexamic acid in marketed tablets in Table 4 show that all of the tablets examined on the market contain tranexamic acid, as evidenced by an overall similarity index value of more than 0.90. The similarity index is a value that indicates the similarity between the unknown sample and the known standard in the system library; the closer to 1.00 the similarity index is, the more similar the samples and standards [35]. A good similarity index value is not less than 0.80, indicating the similarity between unknown samples and standards in the system library [36].

The quantitative analysis of tranexamic acid in marketed tablets findings in Table 4 shows that all tablets on the market satisfied the standards of the tranexamic acid tablet monograph, as evidenced by overall values ranging from 97.0 to 103.0%. Pharmaceutical preparation distributed in the market is declared good quality if it meets the monograph requirements listed in the compendial both qualitatively and quantitatively [37]. The *Indonesian Pharmacopoeia* includes tranexamic acid tablet monographs that require tranexamic acid levels in tablets to be no less than 95.0% and no more than 105.0 percent of the quantity specified on the label [38].

### 3.4. Validation of the Infrared Spectrophotometry Method

The validation of the developed analysis method by infrared spectrophotometry for qualitative analysis and quantitative analysis of tranexamic acid aims to prove and ensure that the validity of the analysis method has been suitable for its purpose [39]. To obtain valid data, an analyst must pay attention to the processes and procedures carried out during the analysis and validate the analytical methods before applying them to routine analysis. The validation of the analytical method is the final stage that determines the quality of the data generated from an analytical method [40].

The analytical method was validated with parameters of accuracy, precision, detection limit, quantitation limit, linearity, and range. Validation of accuracy parameters using a recovery percentage resulted in a value of 99.88%. These results meet the requirements of the accuracy test with the requirements of acceptance for a recovery percentage between 99% and 101%, indicating that the method developed has good accuracy [41]. The validation of precision parameters using the relative standard deviation resulted in a value of 0.50%. These results meet the requirements of the precision test with the requirements of acceptance for a relative standard deviation less than 2%, indicating that the method developed has good precision [42].

The validation of detection limit parameters and quantitation limit parameters using the series concentration and the series area from the regression data set resulted in respective values of 0.0079% and 0.0239%. These values are much lower than the target analysis concentration of 0.50% (the detection limit concentration obtained is 63 times lower, and the quantitation limit concentration obtained is 21 times lower than the concentration). The lower the detection and quantitation limit concentration, the better the method developed [43] and the higher the sensitivity [44].

The validation of linearity parameters using the coefficient of correlation (R) resulted in a value of 0.9997. These results meet the requirements of the linearity test with the requirements of acceptance for the correlation coefficient of more than 0.99, indicating that the method developed has good linearity (good relationship between concentration abscissa, X, and area ordinate, Y) [45]. The validation of the range parameter gives a concentration range of 0.40% to 0.60%. The range of an analytical method is the interval between the lower limit and the upper limit of the analyte concentration in an analytical approach that meets the appropriate requirements for accuracy, precision, and linearity [46].

The qualitative analysis indicates the similarity of the infrared spectrum of the tranexamic acid in marketed tablets with the infrared spectrum of the standard tranexamic acid. The quantitative analysis indicates that the concentration of tranexamic acid in marketed tablets depends on the area of the tranexamic acid standard. The regression model indicates the level of determination and the level of a good relationship between concentration and area. The infrared spectrophotometric method that was successfully developed is specifically for the qualitative analysis and quantitative analysis of tranexamic acid in marketed tablet preparation. Reasonable specificity is shown from regression, qualitative analysis, and quantitative analysis [47].

## 4. Materials and Methods

### 4.1. Tools and Materials

This research used the following tools and materials: Fourier transform infrared spectrophotometer with zinc selenium as a beam splitter and a transmission module for a potassium bromide pellet holder (Agilent-Cary 630, Yishun, Singapore), personal computer (Lenovo, Beijing, China), printer (Canon, Tokyo, Japan), analytical balance (Precisa-LX 320, Dietikon, Switzerland), pellet press (Specac-Hanheld, Orpington, United Kingdom), porcelain mortar and pestle (Merck, Jakarta, Indonesia), agate mortar and pestle (Merck, Jakarta, Indonesia), glassware (Iwaki, Jakarta, Indonesia), Microlab (Agilent–OQ, PC, Quant, Lite, Yishun, Singapore), statistical package for the social sciences (International Business Machines Corporation, Selangor, Malaysia), tranexamic acid (Merck, Jakarta, Indonesia), potassium bromide (Merck, Jakarta, Indonesia), Transamin^®^ 500 mg tablet (Otto Pharmaceutical Industries, Bandung, Indonesia), Plasminex^®^ 500 mg tablet (Sanbe Farma, Bandung, Indonesia), Pytramic^®^ 500 mg tablet (Pyridam Farma, Cianjur, Indonesia), Kalnex^®^ 500 mg tablet (Kalbe Farma, Jakarta, Indonesia), Nexa^®^ 500 mg tablet (Sanbe Farma, Bandung, Indonesia), Nexitra^®^ 500 mg tablet (Ifars, Solo, Indonesia), tranexamic acid 500 mg tablet (First Medifarma, Sidoarjo, Indonesia), and tranexamic acid 500 mg tablet (Bernofarm, Sidoarjo, Indonesia).

### 4.2. Preliminary Study of Infrared Spectrophotometry

The preliminary study of tranexamic acid uses the Kotadiya and Khristi [48] method with minor modifications in the weight and concentration. Tranexamic acid standard and a potassium bromide mixture was prepared to obtain the final concentration of 0.5% tranexamic acid in potassium bromide with a gradual dilution using the following procedure: weighing 13.0 mg of tranexamic acid, weighing 987.0 mg of potassium bromide, mixing into the agate mortar, grinding the mixture with agate pestle until it was homogeneous (obtained initial mixture with a concentration of tranexamic acid of 1.3% in potassium bromide); weighing 38.5 mg of the initial mix; weighing 61.5 mg of potassium bromide; mixing into the agate mortar; grinding with agate pestle until mixed homogeneously (obtained final mixture with a concentration of 0.5% of tranexamic acid in potassium bromide); weighing the final mixture in the amount of 40.0 mg put into the pellet press; pressing at a pressure of 2.0 tons for 2.0 min; measuring the spectrum using an infrared spectrophotometry equipped with a transmission module, connected to the personal computer and printer, with the Microlab application installed; analyzing the pellet at wave numbers 4000 to 650 cm^−1^; measuring the background with the potassium bromide pellet; analyzing the potassium bromide pellet and mixture of tranexamic acid with a potassium bromide pellet to obtain the specific wavenumbers of tranexamic acid for qualitative and quantitative analyses.

### 4.3. Regression of the Tranexamic Acid Standard

The simple linear regression of tranexamic acid uses the Singh et al. [49] method with minor modifications in the weight and concentration. A tranexamic acid standard and potassium bromide mixture regression data set was prepared to obtain the final concentration from 0.00% to 0.50% tranexamic acid in potassium bromide with a gradual dilution using the following procedure: weighing 13.0 mg of tranexamic acid, weighing 987.0 mg of potassium bromide, mixing into the agate mortar, and grinding with agate pestle until homogeneous (obtained initial mixture with a concentration of tranexamic acid of 1.3% in potassium bromide); weighing the initial mixture of 30.8, 34.7, 38.5, 42.4, and 46.2 mg; weighing the potassium bromide of 69.2, 65.3, 61.5, 57.6, and 53.8 mg, mixing into the agate mortar, grinding with agate pestle until mixed homogeneously (obtained final mixture with a concentration of 0.40%, 0.45%, 0.50%, 0.55%, and 0.60% of tranexamic acid in potassium bromide); weighing the final mixture in the amount of 40.0 mg put into the pellet press; pressing at a pressure of 2.0 tons for 2.0 min; measuring the spectrum using an infrared spectrophotometer equipped with a transmission module, connected to the personal computer and printer, and with a Microlab application installed and a statistical package for the social sciences application; analyzing the pellet at wave numbers 4000 to 650 cm^−1^; measuring the background with the potassium bromide pellet; analyzing the potassium bromide pellet and the regression data set mixture of tranexamic acid with a potassium bromide pellet to obtain the regression equation and coefficient of determination.

### 4.4. Analysis of Tranexamic Acid in Marketed Tablets

The analysis of tranexamic acid in marketed tablets uses the method from Sheeja and Swapna [50] with minor modifications in the weight and concentration. The mixture of tranexamic acid in marketed tablets and potassium bromide was prepared to obtain the final concentration of 0.5% tranexamic acid in potassium bromide with a gradual dilution using the following procedure: weighing 20 tablets, inserting them in a porcelain mortar, and grinding them using a porcelain pestle until they became a homogeneous powder; weighing an amount of the powder equivalent to 13.0 mg of tranexamic acid; weighing 987.0 mg of potassium bromide, mixing into the agate mortar, and grinding it with the agate pestle until it was homogeneous (obtained initial mixture with concentration of tranexamic acid of 1.3% in potassium bromide); weighing 38.5 mg of the initial mix, weighing 61.5 mg of potassium bromide, mixing all ingredients into the agate mortar, and grinding with an agate pestle until mixed homogeneously (obtaining the final mixture with a concentration of tranexamic acid of 0.5% in potassium bromide); weighing the final mixture in the amount of 40.0 mg put into the pellet press; pressing at a pressure of 2.0 tons for 2.0 min; measuring the spectrum by using an infrared spectrophotometer equipped with a transmission module, connected to the personal computer and printer, with a Microlab application installed; analyzing the pellet at wave numbers 4000 to 650 cm^−1^; measuring the background with the potassium bromide pellet; analyzing qualitatively samples of tranexamic acid tablets with the similarity index (similarity of sample spectrum to standard spectrum); analyzing quantitatively samples of tranexamic acid tablets by calculating the level of tranexamic acid in the sample with a predetermined concentration of tranexamic acid using a regression equation.

### 4.5. Validation of the Infrared Spectrophotometry Method

The validation of the analysis method was based on the method from Judeh et al. [51] and Naz et al. [52], with minor modifications in weight and concentration. Validation includes accuracy, precision, detection limit, quantitation limit, linearity, and range. The accuracy of the analysis method with the recovery percentage parameter was carried out using a standard addition method for three specific concentrations (80%, 100%, and 120%). Each specific concentration contained a 30% standard added to a 70% analyte for three replications, then was analyzed using the same technique as in a sample analysis. The precision of the analysis method with the relative standard deviation parameter against the data obtained from three specific concentrations and three replications for each specific concentration. The linearity of the analysis method with the coefficient of the correlation parameter was determined using data obtained from the regression data set. The detection limit and the quantitation limit of the analysis method were determined using the data obtained from the regression data set based on the standard deviation of the response and the slope. The range of the analysis method is determined from the upper and lower limits of concentration that meet the criteria of accuracy, precision, and linearity [53]. The specificity of the analysis method was determined from the regression data set and qualitative and quantitative analyses of the sample [54].

## 5. Conclusions

The infrared spectrophotometry method has been successfully developed for the analysis of tranexamic acid. It has been applied for qualitative analysis and quantitative analysis of tranexamic acid in marketed tablets to ensure that all marketed tablets meet the qualitative and quantitative requirements of the tablet monograph stated in the *Indonesian Pharmacopoeia*, 6th Edition, 2020. The infrared spectrophotometry method has been verified and shown to be accurate, precise, detectable, quantifiable, linear, range, and specific.

## Figures and Tables

**Table 1:** Reported previous analyses of tranexamic acid.

Method	Detector	Treatment
Spectrophotometry	Ultraviolet Visible [10]	2,6-Dichloroquinone-4-Chlorimide [10]
	Infrared [11]	First Order [11]
High-Performance Liquid Chromatography	Mass Spectrophotometry [13]	[13]
	Evaporative Light Scattering [14]	[14]
	Ultraviolet-Visible Detector [12]	Benzene Sulfonyl Chloride [12]
	Photo Diode Array [15]	[15]

**Table 2:** Specific wavenumbers and functional groups of tranexamic acid.

Obtained Wavenumber	Reference Wavenumber	Functional Group/Fingerprint
2965.11 cm^−1^	±3000 cm^−1^ (Strong & Board)	–COOH (O–H Stretching)
769.73 cm^−1^	600 cm^−1^ to 800 cm^−1^ (Medium)	–COOH (O–H Bending)
1162.86 cm^−1^ and 1196.50 cm^−1^	1000 cm^−1^ to 1200 cm^−1^ (Medium)	–COOH (O–H Bending)
1569.18 cm^−1^ and 1541.34 cm^−1^	1300 cm^−1^ to 1600 cm^−1^ (Medium)	–COOH (O–H Bending)
1638.25 cm^−1^	<1700 cm^−1^ (Medium)	–COOH (C=O Stretching)
1010.16 cm^−1^ and 922.53 cm^−1^	±1050 cm^−1^ (Medium)	–COOH (C–O Stretching)
3445.97 cm^−1^	3650–3250 cm^−1^ (Medium)	–NH_2_ (N–H Stretching)
1638.22 cm^−1^	1650–1590 cm^−1^ (Medium)	–NH_2_ (N–H Bending)
1030.64 cm^−1^	1090–1020 cm^−1^ (Medium)	–NH_2_ (C–N Stretching)
2715.39 cm^−1^ and 2611.01 cm^−1^	>3000 cm^−1^ (Medium)	Alkane (C–H Stretching)
2935.05 cm^−1^	±2935 cm^−1^ (Medium)	Alkane (C–H Stretching)
2879.43 cm^−1^ and 2868.32 cm^−1^	±2860 cm^−1^ (Medium)	Alkane (C–H Stretching)
2210.28 cm^−1^	±2200 cm^−1^ (Medium)	Alkyne (C≡C Stretching)
1453.69 cm^−1^ and 1384.70 cm^−1^	±1470 cm^−1^ (Medium)	Alkane (C–H Bending)
702.57 cm^−1^	±720 cm^−1^ (Medium)	Alkane (C–H Bending)

**Table 3:** Series concentration and series area from the regression data set series of tranexamic acid.

Number	Concentration (%)	Area (AU)
1	0.00	0.2444308000
2	0.40	125.8313214400
3	0.45	142.4944207150
4	0.50	158.3252190500
5	0.55	169.8243798000
6	0.60	186.2427745000

**Table 4:** Results for qualitative analysis and quantitative analysis of tranexamic acid in marketed tablets.

Number	Sample	Qualitative Analysis (Tranexamic Acid Similarity Index)	Quantitative Analysis (Tranexamic Acid Level)
1	Transamin^®^ 500 mg Tablet (Otto Pharmaceutical Industries)	0.9083	97.56% ± 0.19%
2	Plasminex^®^ 500 mg Tablet (Sanbe Farma)	0.9157	101.80% ± 0.24%
3	Pytramic^®^ 500 mg Tablet (Pyridam Farma)	0.9065	99.73% ± 0.22%
4	Kalnex^®^ 500 mg Tablet (Kalbe Farma)	0.9279	102.74% ± 0.27%
5	Nexa^®^ 500 mg Tablet (Sanbe Farma)	0.9189	100.47% ± 0.22%
6	Nexitra^®^ 500 mg Tablet (Ifars)	0.9192	99.50% ± 0.21%
7	Tranexamic Acid 500 mg Tablet (First Medifarma)	0.9027	98.65% ± 0.21%
8	Tranexamic Acid 500 mg Tablet (Bernofarm)	0.9122	102.55% ± 0.26%

## Data Availability

The data presented in this study are available in this article.

## Sample Availability

Samples are available from the authors.
